# Supplementary material for: Synthesis of Bio-Based Polyurethanes from Functionalized Sunflower Seed Oil
Source: Int J Mol Sci. 2025 Nov 25;26(23):11380. doi: 10.3390/ijms262311380 (PMC12692041; doi:10.3390/ijms262311380)
Supplement: Supplementary file 1 [file ijms-26-11380-s001.zip › ijms-3984393-supplementary.pdf]

Synthesis of Bio-Based Polyurethanes from Functionalized Sunflower Seed Oil

Csilla Lakatos[1], Katalin Czifrák[1], Csaba Cserháti[2], Réka Borsi-Gombos[3], Lajos Nagy[1], Miklós Zsuga[1] and Sándor Kéki[1*]

[1] Department of Applied Chemistry, University of Debrecen, Egyetem tér 1, H-4032 Debrecen, Hungary;

[2] Department of Solid State Physics, University of Debrecen, Bem tér 18/b, H-4026 Debrecen, Hungary

[3] Department of Physical Chemistry, University of Debrecen, Egyetem tér 1, H-4032 Debrecen, Hungary

*Correspondence: keki.sandor@science.unideb.hu; Tel.: +36-52-512-900 (ext. 22455)

Table of contents

Determination of the OH number…………………………………………...……..……...3

Table S1. The possible products formed in the epoxidation reaction of sunflower oil……....4

Table S2. The parameters of eq. 6 determined from fitting………………...……...………6

Table S3. The parameters of eq. 7 determined from fitting…………………………….…....6

Figure S1. MALDI-TOF MS spectrum of sunflower oil…………………….……….……...7

Figure S2. ^{13}C-NMR spectrum of epoxidized oil (EPO)……………………….……………8

Figure S3. MALDI-TOF MS spectrum of epoxidized oil (EPO)……………………..………9

Figure S4. Relaxation time distributions calculated by MATLAB...………………………….10

Figure S5. DSC curve for PTMEG……………………………………………………...11

Figure S6. DMA curves for PUs 1, 3, 5 and 6……………………...……………………..12

Determination of the OH number

The OH numbers of the oil polyols were determined according to the following formula:

$$OH = 56.1 \cdot N \cdot \frac{(V_2 - V_1)}{a} + S$$

where

56.1 = KOH equivalent weight

N = Nominal normality of KOH solution multiplied by a factor

V_2 = Volume of KOH solution consumed during titration of blank sample (cm^3)

V_1 = Volume of KOH solution consumed during titration of sample (cm^3)

a = mass of the sample (g)

S = Acid value of the tested oil

given in Hungarian patent No MSZ 3629

Table S1. The possible products formed in the epoxidation reaction of sunflower oil. In this notation, PA, OA, LA represent the palmitic acid, oleic acid, linoleic acid while EO, OH and DB stand for the epoxy and the hydroxyl groups and the double bonds, respectively.

Starting nominal m/z	Number of PA	Number of OA	Number of LA	Number of EO	Number of OH	Number of DB	Observed m/z
877	1	0	2	2	0	2	909.72
877	1	0	2	0	2	3	911.73
879	1	1	1	2	0	1	
879	1	1	1	0	2	2	913.75
881	1	2	0	2	0	0	
877	1	0	2	3	0	1	925.71
877	1	0	2	1	2	2	927.73
879	1	1	1	3	0	0	
901	0	0	3	0	2	5	935.73
903	0	1	2	2	0	3	
903	0	1	2	0	2	4	937.75
905	0	2	1	2	0	2	
905	0	2	1	0	2	3	939.76
907	0	3	0	2	0	1	
877	1	0	2	4	0	0	941.71
907	0	3	0	0	2	2	941.78
877	1	0	2	2	2	1	943.72
877	1	0	2	0	4	2	945.74
879	1	1	1	2	2	0	
901	0	0	3	3	0	3	949.71
881	1	2	0	0	4	0	949.77
901	0	0	3	1	2	4	951.73
903	0	1	2	3	0	2	
903	0	1	2	1	2	3	953.74
905	0	2	1	3	0	1	
905	0	2	1	1	2	2	955.76
907	0	3	0	3	0	0	
901	0	0	3	4	0	2	965.71
901	0	0	3	2	2	3	967.72
903	0	1	2	4	0	1	
901	0	0	3	0	4	4	969.74
903	0	1	2	2	2	2	
905	0	2	1	4	0	0	
903	0	1	2	0	4	3	971.75
905	0	2	1	2	2	1	
905	0	2	1	0	4	2	973.77
907	0	3	0	2	2	0	
901	0	0	3	3	2	2	983.72
903	0	1	2	5	0	0	
901	0	0	3	1	4	3	985.73

Starting nominal m/z	Number of PA	Number of OA	Number of LA	Number of EO	Number of OH	Number of DB	Observed m/z
903	0	1	2	3	2	1	
903	0	1	2	1	4	2	987.75
905	0	2	1	3	2	0	
901	0	0	3	4	2	1	999.71
901	0	0	3	2	4	2	1001.73
903	0	1	2	4	2	0	
901	0	0	3	0	6	3	1003.74
903	0	1	2	2	4	1	
903	0	1	2	0	6	2	1005.76
905	0	2	1	2	4	0	

Table S2. The parameters of eq. 6 determined from fitting.

Sample	C_1	a_1	C_2	a_2	a_3	a_4
PU-1	0.15	2.10	15.36	0.74	1.65	0.30
PU-2	0.40	1.74	13.70	0.53	1.20	0.25
PU-3	6.63	0.65	8100	1.39	7.65	0.27
PU-4	6.42	0.67	29500	1.54	9.06	0.26
PU-5	0.20	2.01	13.89	0.49	1.06	0.23
PU-6	4.29	0.70	12.55	0.56	1.96	0.75

Table S3. The parameters of eq. 7 determined from fitting.

Sample	τ(s)	λ	A (MPa)
PU-1	12.05	0.32	0.27
PU-2	8.07	0.30	0.29
PU-3	2.63	0.28	0.36
PU-4	3.45	0.25	0.37
PU-5	8.81	0.31	0.30
PU-6	7.53	0.30	0.28

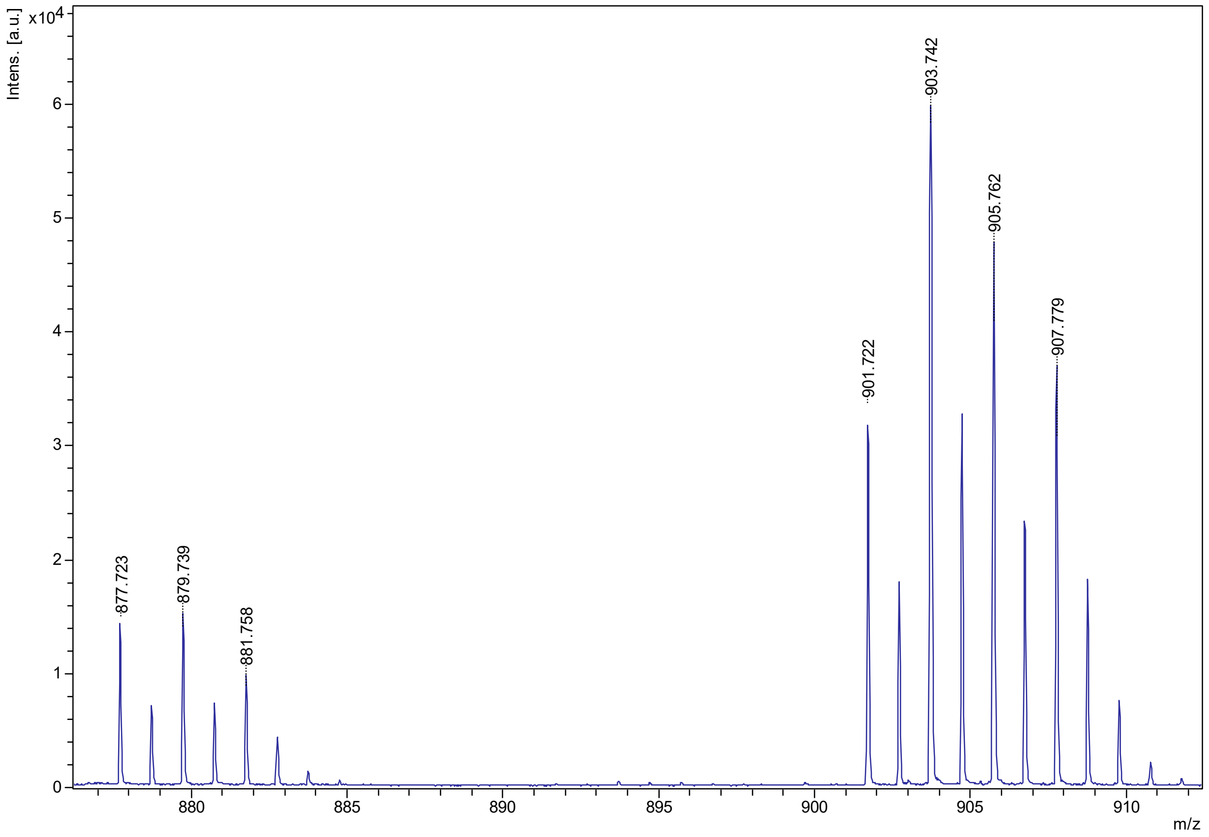

Figure S1. MALDI-TOF MS spectrum of sunflower oil.

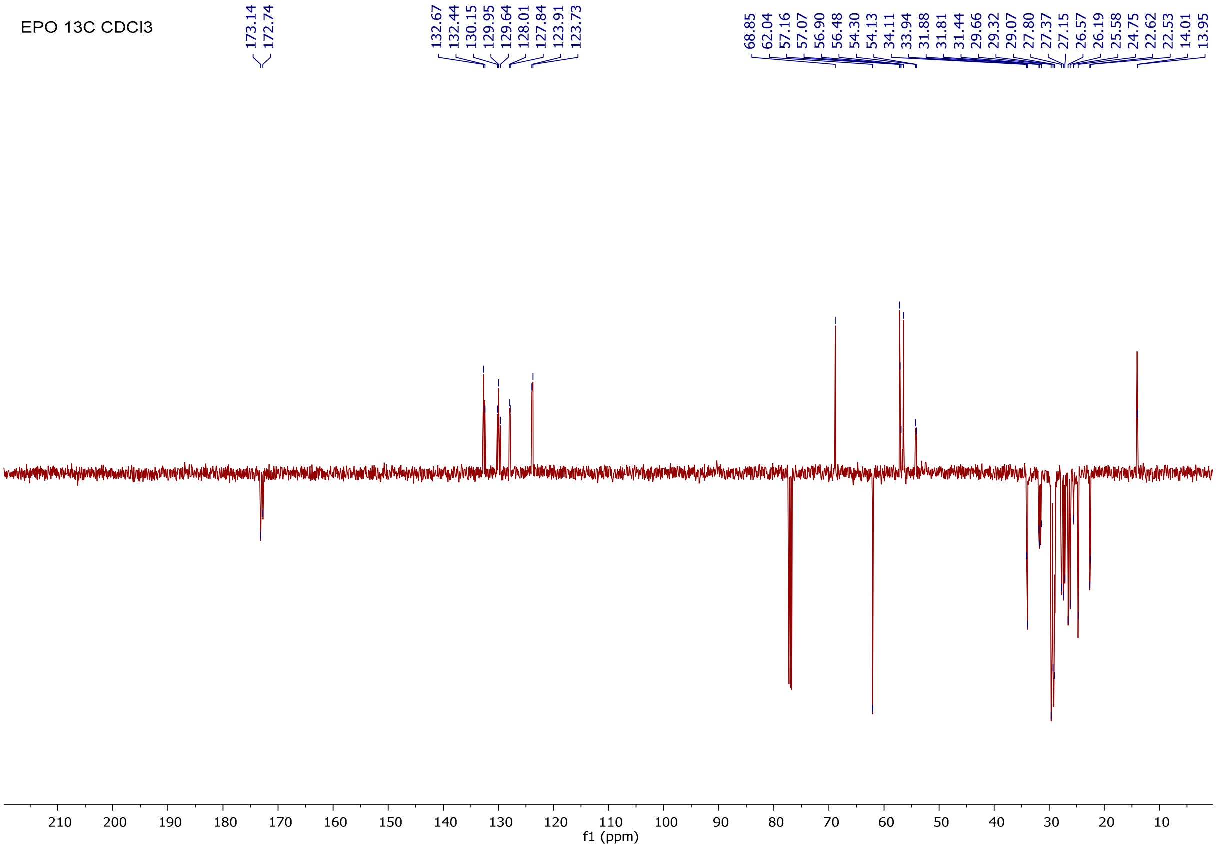

Figure S2. ^{13}C-NMR spectrum of epoxidized oil (EPO).

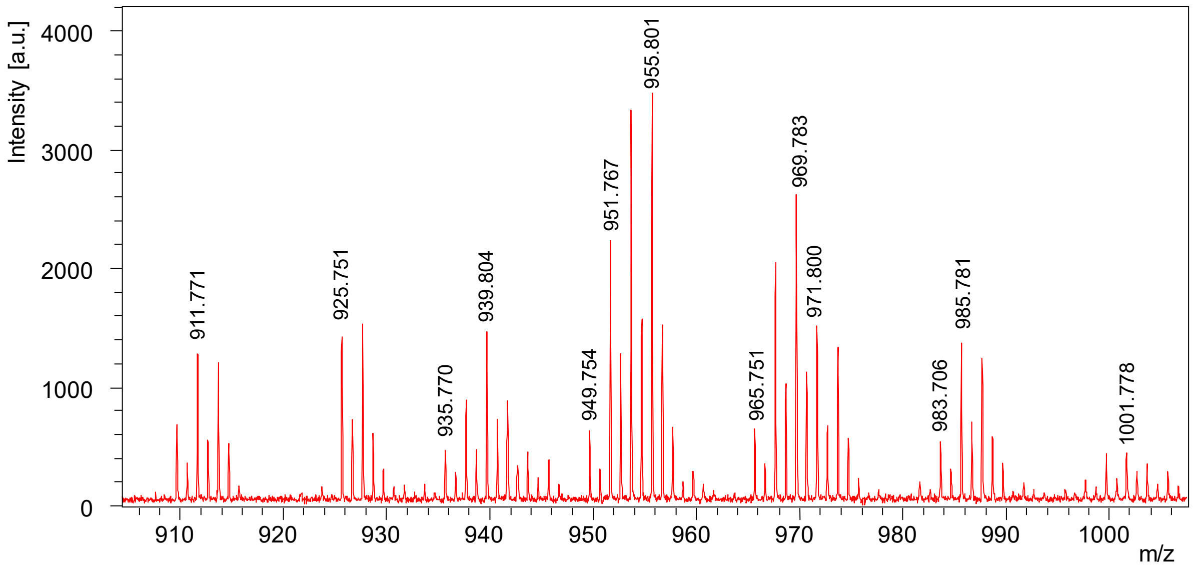

Figure S3. MALDI-TOF MS spectrum of epoxidized oil (EPO).

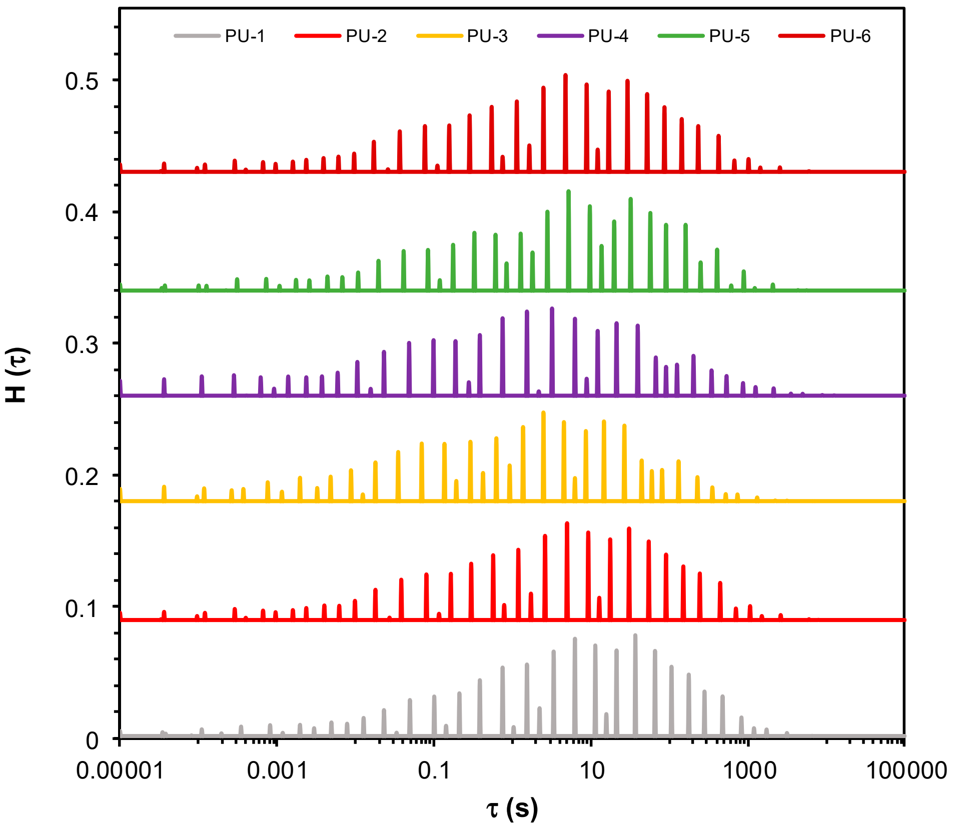

Figure S4. Relaxation time distributions calculated by MATLAB

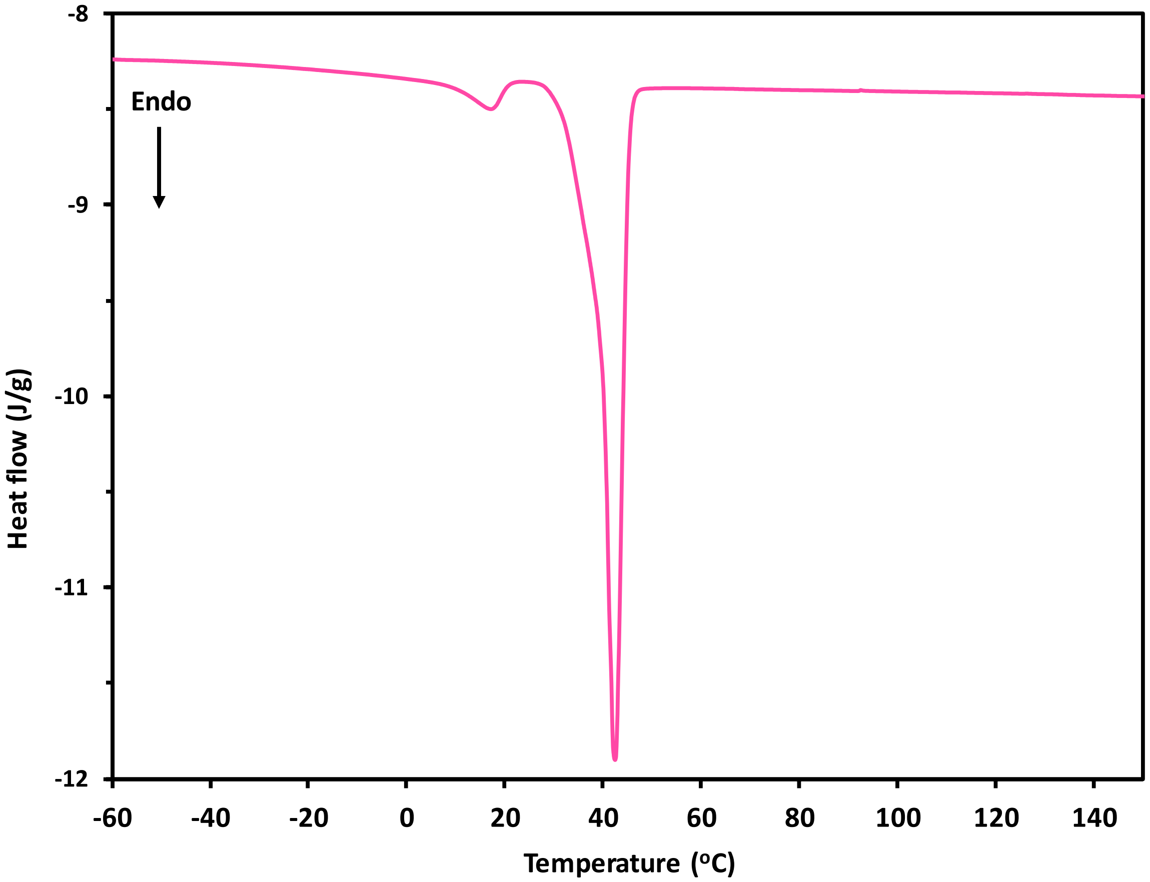

Figure S5. DSC curve for PTMEG.

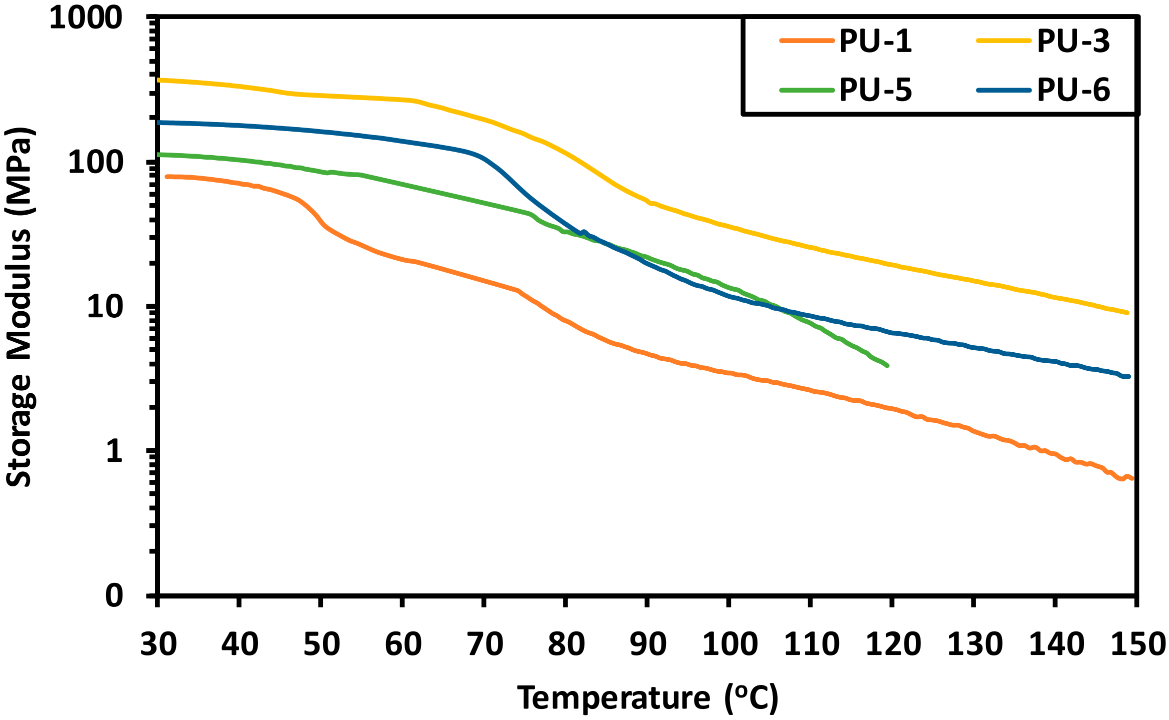

Figure S6. DMA curves for PUs 1, 3, 5 and 6.
